# Improvement of Black-Odor Water by *Pichia* Strain GW1 under Optimized NH_3_-N Degradation Conditions

**DOI:** 10.1155/2020/1537873

**Published:** 2020-02-17

**Authors:** Haiwei Xie, Mingyang Wang, Huayang Zeng, Mingrong Yu, Zijian Wu, Shuhong Chen, Shaotian Zhao, Jie Zheng, Dun Deng

**Affiliations:** ^1^School of Life Science, Huizhou University, Huizhou 516007, China; ^2^Key Laboratory of Zoonosis of Liaoning Province, College of Animal Science & Veterinary Medicine, Shenyang Agricultural University, Shenyang 110866, China; ^3^Ruijie Environmental Protection Engineering Co., Ltd. Huizhou 516007, China; ^4^Institute of Animal Science, Guangdong Academy of Agricultural Sciences, State Key Laboratory of Livestock and Poultry Breeding, Guangzhou 510640, China

## Abstract

In this study, a yeast strain with an outstanding NH_3_-N degradation ability was isolated from the sediment of a black-odor water channel in Guangdong Province, China. Based on phenotypic and phylogenetic analysis, this strain was identified as *Pichia kudriavzevii* GW1. The optimum conditions for NH_3_-N degradation by the GW1 strain were as follows: 0.3% inoculum concentration, 1.5 L/min aeration, pH 7, and a temperature of 35°C. Under optimized conditions, the GW1 strain degraded 95.5% of the NH_3_-N. The strain was then added to simulated black-odor water under optimal degradation conditions to investigate changes to the bacterial community over time. 16S rRNA sequencing of samples collected on days 0, 7, 14, and 21 showed that, in the presence of the GW1 strain, the relative abundances of the phyla Proteobacteria, Bacteroidetes, Chloroflexi, and Firmicutes increased in the black-odor water. In addition, the relative abundance of *Propionivibrio*, a known NH_3_-N degrading genus, increased. This study will facilitate the use of microbiological methods to repair black-odor water.

## 1. Introduction

With increased urbanization and industrialization, a large amount of domestic sewage and industrial wastewater are discharged into rivers, causing black-odor water in urban rivers and lakes. Currently, a total of 1861 water bodies (85.7% of rivers and 14.3% of lakes) in China are categorized as black-odor water [1]. The concentration of manganese and iron ions in black-odor water exceeds the standard guidelines, and suspended matter in the water interacts with organic matter to precipitate at the bottom of the river, causing the black-odor water to further deteriorate. Hydrogen sulfide, amine, methane, and other gases in the water emit an odor and pollute the air [2, 3]. Black-odor water not only poses a threat to human health but also causes substantial pollution to the ecological environment [4, 5]. Currently, black-odor water is treated by physical methods, including dredging of sediments, sediment cover, and artificial aeration; chemical methods, including addition of aluminum salts, sodium nitrate, calcium nitrate, hydrogen peroxide, and organisms; and biological methods, including purification using aquatic plants, microbial fortification and exogenous, microbial transmission [6–8]. Traditional methods are often considered to have low efficiency, poor stability, and high sensitivity to environmental conditions [9–11]. Therefore, it is necessary to treat black-odor water more safely, economically, and efficiently.

Contaminants in water provide carbon sources and energy substrates, or intermediate metabolites, for microbial growth and reproduction to provide essential nutrients for microorganisms, which promotes the secretion of sufficient active enzymes for the degradation of pollutants [12, 13]. The natural attenuation of water pollutants depends on the microbial degradation process. Due to the limitation of nutrient concentrations, pH, redox potential, and temperature in the environment, the microbial degradation process of indigenous microorganisms is slow. By adding functional microorganisms to the water body, the degradation process can be shortened, and pollutants can be quickly removed [14]. The functional microorganisms in microbial agents have high species diversity and the ability to degrade different pollutants [14]. Microorganisms form a commensal community with a complex composition and stable structure and can degrade pollutants by secreting various extracellular or intracellular enzymes [15]. The microbial community can facilitate the decomposition of complex organic matter into simple inorganic substances and the conversion of toxic substances into nontoxic or low-toxic substances; thus, the microorganisms continue to oxidize and decompose pollutants, so that the pollutants in the water body are removed [16–18]. However, the ability of microbial agents to degrade black odorous sewage has not been reported, and the degradation mechanism of microbial agents on black-odor water needs further clarification.

In the present study, our aim was to isolate microorganisms that possess a high degradation ability to treat black-odor water. To that end, we determined the morphology and characteristics of isolated bacterial strains and investigated their ability to improve the removal rates of COD and ammonia nitrogen (NH_3_-N) in black-odor water. In addition, we constructed a composite fungicide through the selected strains. We then identified the optimal conditions of composite microbial agents and examined their ability to treat black-odor water. The results of this work can provide an effective way to treat black-odor water.

## 2. Materials and Methods

### 2.1. Sampling

**Figure 1:**
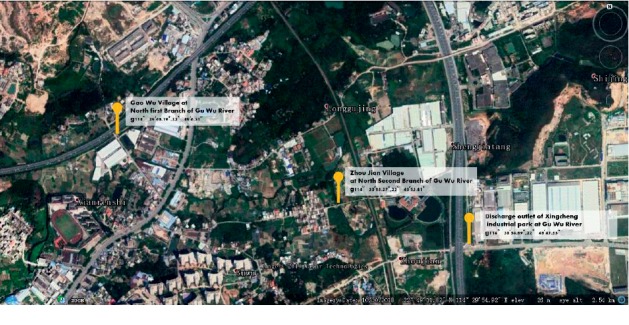
Collection site of black-odor water and river sediment. Latitude and longitude of sampling locations: (1) E:114.494550, N:22.818385; (2) E:114.504243, N:22.814963; and (3) E:114.509975, N:22.812971.

River sediments and black odorous water samples were collected from a river channel in Huizhou City, Guangzhou Province, China (Figure 1). The river is classified as black-odor water. Surface sediments (10–15 cm) from the six sampling points of the two river sections were collected, sealed in a sterile bag, and then stored in a refrigerator at 4°C.

### 2.2. Culture Media

Fungi were grown in Martin's medium (pH 7.4–7.6), containing potassium dihydrogen phosphate (1 g/L), magnesium sulfate (0.5 g/L), peptone (5 g/L), glucose (10 g/L), agar (19 g/L), Bengal red (0.033 g/L), chloramphenicol (0.1 g/L), and distilled water (1000 mL). Bacteria were grown in beef extract peptone medium containing beef extract (3 g/L), peptone (10 g/L), sodium chloride (5 g/L), starch (1 g/L), agar (15 g/L), and distilled water (1000 mL). Actinomycetes were grown in Gao's No.1 medium (pH 7.4–7.6) containing soluble starch (20 g/L), potassium dihydrogen phosphate (0.5 g/L), sodium chloride (0.5 g/L), magnesium sulfate (0.2 g), potassium nitrate (1 g/L), ferrous sulfate (0.01 g/L), agar (15 g/L), 3% potassium dichromate (3.3 mL), and distilled water (1000 mL).

### 2.3. Enrichment and Screening of Microorganisms for NH_3_-N-Degrading Ability

Triplicate river sediment samples (10 g each) were added to sterilized 150 mL conical flasks containing 100 mL beef extract peptone medium, Gao's No. 1 medium, or Martin's liquid medium. Flasks were placed in a shaker and incubated for 2 days at a constant temperature (28°C). After the culture medium became turbid, 10 mL of the enrichment was aliquoted into an Erlenmeyer flask, supplemented with 100 mL of fresh medium, returned to the shaker, and incubated for 2 days at constant temperature (28°C). This process was repeated 2-3 times. Using this method, bacteria, fungi, and actinomycetes in the river sediment were separately enriched.

About 100 *μ*L of different concentrations (10^−1^, 10^−2^, 10^−3^, 10^−4^, and 10^−5^) of bacteria, fungi, and actinomycetes suspension was taken and inoculated into the prepared medium (beef extract peptone agar medium, Martin's agar medium, and Gao's No.1 agar medium). After culturing for 2 days in a constant temperature incubator at 30°C, dominant colonies with different characteristics were picked and grown on the selection medium. After inoculating the bacteria, the selected medium was placed in a constant temperature incubator at 30°C for 2 days. Microorganisms capable of degrading NH_3_-N in black odorous sewage were initially screened.

Isolated strains were cultured in screening medium (30°C, 120 r/min). A negative control was performed by incubating screening medium without inoculum. NH_3_-N was measured using the Nessler colorimetry method [19]. The NH_3_-N degradation rate of each strain was determined and strains with higher degradation rates were selected for identification.

### 2.4. Phenotypic Characterization of the Optimal Bacteria

The GW1 strain was subjected to Gram staining, morphological analysis, and 18S rDNA sequencing. Genomic DNA was isolated using the Ezup Column Bacteria Genomic DNA Purification Kit (Sangon, Beijing, China). The standard 18S rDNA gene PCR primers (ITS1: TCCGTAGGTGAACCTGCGG and ITS4: TCCTCCGCTTATTGATATGC) were used to amplify the target gene sequence of the GW1 strain. The PCR cycles were as follows: 2 min at 98°C; followed by 25 cycles of 98°C (10 s), 55°C (15 s), and 72°C (15 s); and a final extension at 72°C for 5 min. The resulting PCR products were then sequenced by Tsingke (Beijing, China). Sequences similar to the GW1 strain were identified using BLAST (http://www.ncbi.nlm.nih.gov). All similar sequences were retrieved from NCBI and a phylogenetic tree was constructed using DNAStar.

### 2.5. Optimization of NH_3_-N Degradation by the GW1 Strain

The natural black-odor water environment was simulated by adding approximately 5 cm of the river bottom mud into an 8 L glass cylinder containing approximately 4 L of black-odor sewage. The cylinder was placed at room temperature (25°C), with light and other factors remaining consistent. The effects of concentration, aeration, pH, and temperature were evaluated to determine the optimal conditions for NH_3_-N degradation using the simulated black-odor water environment. In order to evaluate the effect of the GW1 strain concentration on the degradation of NH_3_-N, the GW1 strain was added to the simulated black-odor water environment at concentrations of 0.05%, 0.1%, 0.2%, and 0.4%. A control without the addition of the GW1 strain was also performed. In order to evaluate the effect of aeration on the ability of the GW1 strain to degrade NH_3_-N, an adjustable aeration pump (maximum aeration volume of 4 L/min) was used to increase the dissolved oxygen content of the different treatments. Five black-odor water aeration levels (0.5 L/min, 1 L/min, 2 L/min, 4 L/min, and 0 L/min (anaerobic control)) were tested. In order to evaluate the effect of pH on the degradation of NH_3_-N by fungi, hydrochloric acid (0.1%) and sodium hydroxide (0.1%) were used to adjust the initial pH of the water to 5, 6, 7, 8, and 9. The pH of the raw water was 6.4. The black-odor water environment without pH adjustment was used as a control group. The temperature most suitable for the degradation of NH_3_-N was determined by incubating treatments at 20°C, 25°C, 30°C, 35°C, and 40°C. For all of the treatments, each group was evaluated in triplicate and the water quality index was measured every 2 days. The degradation rate of black odorous NH_3_-N was detected along with the water quality index. The national standard method was used to detect NH_3_-N and COD in the black-odor water samples. The COD of the water samples was detected using the dichromate method. The NH_3_-N in the sewage sample was detected by Nessler spectrophotometry.

The best three levels for orthogonal experiments are based on single-factor experiments. An orthogonal design containing four factors and three levels was adopted to further optimize the NH_3_-N degradation conditions. The concentration, aeration, pH, and temperature were set at 0.1%, 0.2%, and 0.3%; 0.5 L/min, 1 L/min, and 1.5 L/min, 6.5, 7, and 7.5; and 30°C, 35°C, and 40°C, respectively.

### 2.6. Effect of GW1 Strain on Microorganisms in Black-Odor Water

A black-odor sewage degradation test was carried out under the optimal degradation conditions. DNA was extracted after the addition of the GW1 strain on days 0, 7, 14, and 21 (sample IDs: CON, Aw7, Aw14, and Aw21, respectively) using the E.Z.N.A. Stool DNA Kit (D4015, Omega, Inc., USA) according to the manufacturer's instructions. Nuclease-free water was used as a negative extraction control. Total DNA was eluted with 50 *μ*L of elution buffer and stored at −80°C until being sent to LC-Bio Technology Co., Ltd (Hang Zhou, Zhejiang Province, China) for PCR amplification. The degradation rate of black odorous sewage NH_3_-N and the water quality index were measured.

### 2.7. Statistical Analysis

The data was analyzed using SPSS 20.0 statistical software.

## 3. Results

### 3.1. Screening of NH_3_-N-Degrading Strains

**Figure 2:**
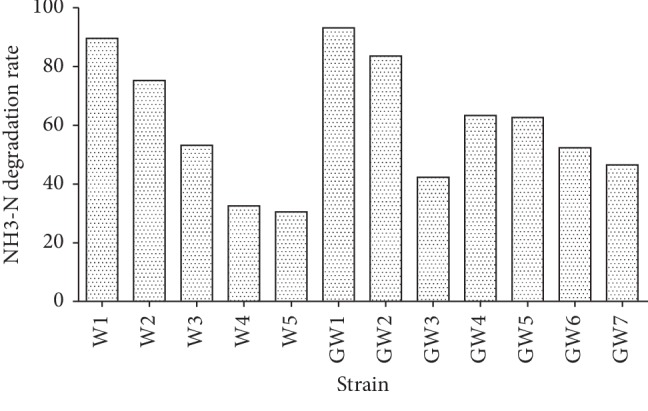
Strains with NH_3_-N-degrading ability.

The screening experiment resulted in the isolation of 12 strains with NH_3_-N degrading abilities from the black-odor sewage.  Table 1 shows the growth of 12 strains in 3 media. Their NH_3_-N (1 g/L) degradation rates ranged from 30.5% to 93.2% (Figure 2) after incubation for two days. The GW1 strain exhibited the highest degradation rate and was selected for further investigation.

### 3.2. Identification of the GW1 Strain

**Figure 3:**
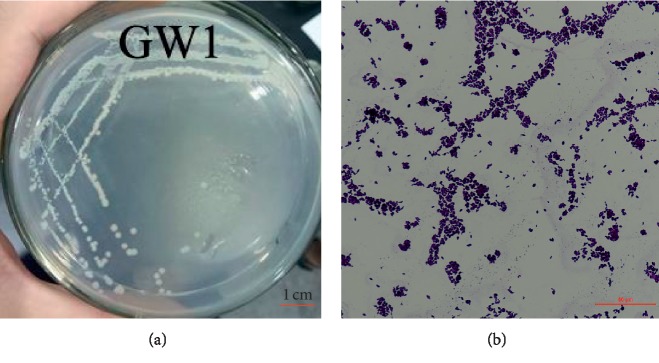
Morphological characteristics of the GW1 strain. (a) Colonies of the GW1 strain on LB agar plate; (b) a photograph of the Gram stain (10 × 60).

The colony of the GW1 strain was smooth-faced, white-colored, and circular with a tidy margin (Figure 3(a)). This baseline was observed under a microscope after Gram staining (Figure 3(b)).

**Figure 4:**
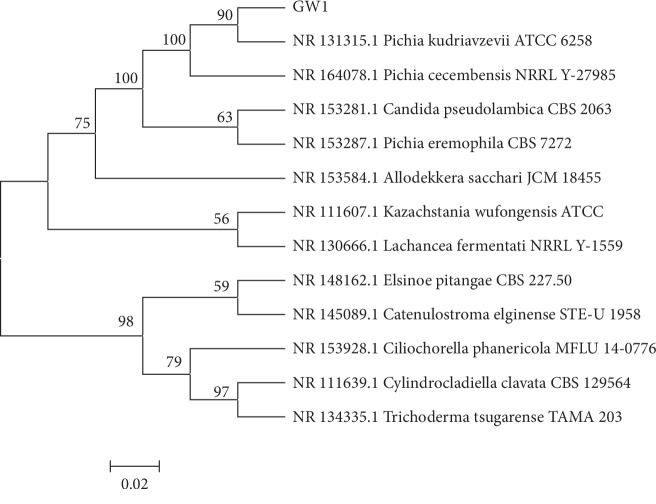
Homology analysis based on partial 18S rDNA sequences of the GW1 strain and the related microorganisms.

DNA sequencing results showed that the obtained 18S rDNA sequence of the GW1 strain was highly homologous to those of *Pichia* strains. A phylogenetic tree (Figure 4) was constructed with similar 18S rDNA sequences using the neighbor-joining method [20]. The results show that the GW1 strain was most closely related to KU962038.1_*Pichia_kudriavzevii*. Based on the morphological, biochemical, and physiological properties, as well as the phylogenetic analysis of the 18S rDNA sequences, the GW1 strain was identified as a *Pichia* strain.

### 3.3. Optimization of the Conditions for NH_3_-N-Degradation

**Figure 5:**
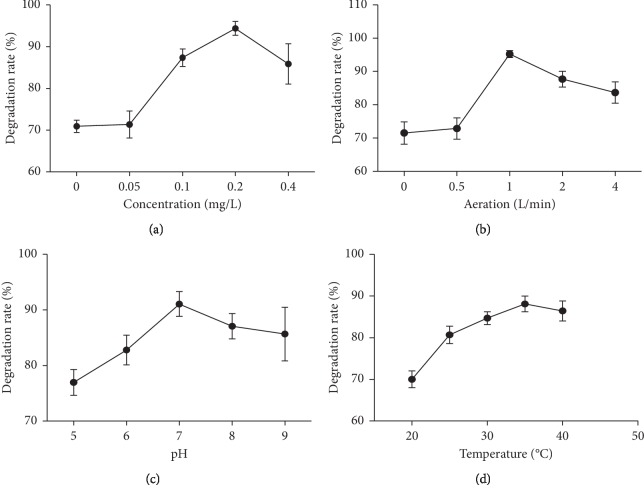
Optimization of the conditions for NH_3_-N degradation. The optimized conditions included compound concentration (a), aeration (b), pH (c), and temperature (d).

The effects of concentration, aeration, pH, and temperature on the degradation of NH_3_-N by the GW1 strain were studied. When GW1 concentration was 0.2% mg/L, the degradation of NH_3_-N was the best (Figure 5(a)).  Figure 5(b) shows the relationship between the aeration rate and NH_3_-N degradation rate. When the aeration rate reached 1 L/min, the degradation rate of NH_3_-N reached the maximum (96.83%), but the degradation rate of NH_4_ decreased with the increase in aeration rate. When the pH was 7, the degradation rate of NH_3_-N reached 92.54%. The degradation rate of NH_3_-N decreased when the pH was increased or decreased.  Figure 5(d) shows the relationship between temperature and NH_3_-N degradation rate. At 35°C, the NH_3_-N degradation rate reached the highest value (88%). When the GW1 strain was treated with black-odor water, the factors affecting both the NH_3_-N and COD removal rates were as follows: *A* > *D*> *B* > *C* (Tables 2 and 3), namely, concentration > temperature > aeration > pH. Therefore, the optimal process is *A*3*B*3*C*3*D*2, specifically the concentration is 0.3%, the aeration is 1.5 L/min, the pH is 7, and the temperature is 35°C. Since the optimal combination was not in the 9-group test, a confirmatory test was performed under these conditions. The removal rate was 95.5%, which was greater than the maximum removal rate in the orthogonal test, indicating that the method is reproducible.

For the factors affecting the COD removal rate, the primary and secondary order is *D* > *B* > *A* > *C*, namely, temperature > aeration > concentration > pH. Therefore, the best process is *A*2*B*3*C*2*D*2, specifically the concentration is 0.2%, the aeration rate is 1.5 L/min, the pH is 7, and the temperature is 35°C. Since the optimal combination was not in the 9-group test, a confirmatory test was performed under these conditions and the GW1 strain was measured. The COD removal rate was 92.7%, which was greater than the maximum removal rate in the orthogonal test, indicating that the method has good repeatability.

### 3.4. Effect of GW1 Strain on Microorganisms in Black-Odor Water

**Figure 6:**
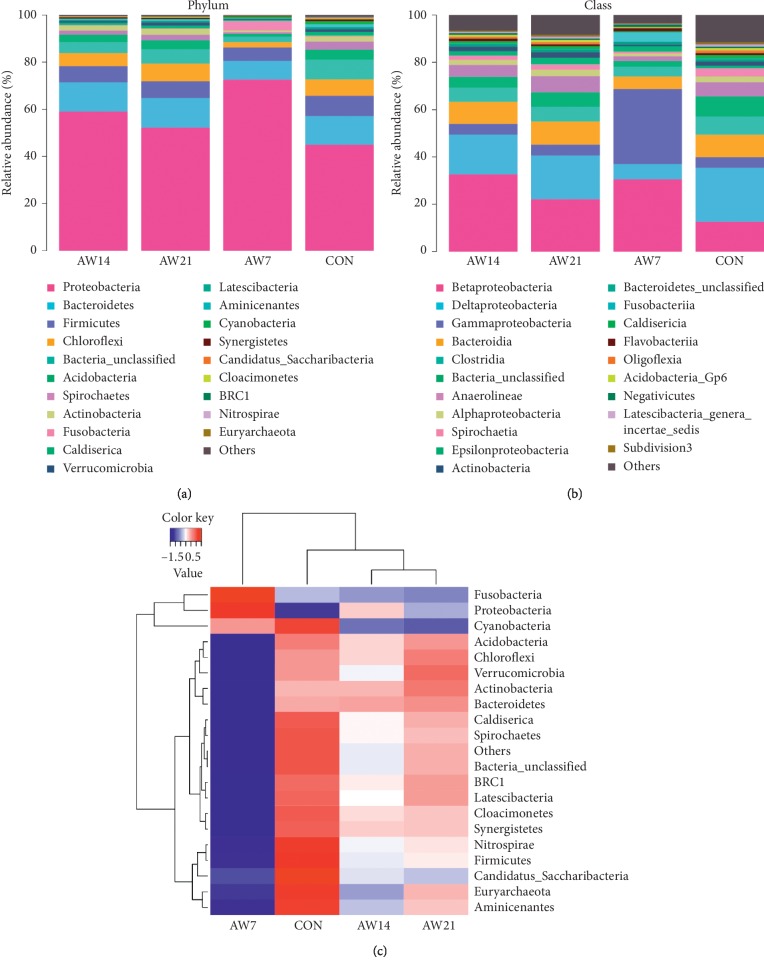
(a) Frequency of the major phyla in each sample. (b) Frequency of the major classes in each section of the sample. (c) The relative abundances of the major bacterial genera (20 are most dominant in each section of every sample).

The relative abundance of bacteria at the phylum level of the four samples is shown in Figure 6(a). Proteobacteria, Bacteroidetes, Chloroflexi, and Firmicutes were the main phyla in all samples; however, their relative abundances were different in each sample. Proteobacteria in the Aw 7, Aw 14, and Aw 21 samples was more abundant than that in the CON sample. The Bacteroidetes and Chloroflexi abundances in the Aw 7 samples were lower compared to the CON sample, while their abundances in the Aw 14 and Aw 21 samples were consistent with the CON sample.

At the class level, the bacterial community was dominated by Betaproteobacteria, Deltaproteobacteria, Gammaproteobacteria, and Bacteroidia (Figure 6(b)). Betaproteobacteria in the Aw 7, Aw 14, and Aw21 samples was more abundant than that in the CON sample. The abundance of Deltaproteobacteria in the Aw 7 sample was lower compared to the CON, Aw 14, and Aw 21 samples. The abundance of Gammaproteobacteria in the Aw 7 sample was higher compared to the CON, Aw 14, and Aw 21 samples. It is clear that the abundances of the dominant flora in the Aw 7 sample were notably different than the other sample.

The heatmap shows the 20 most dominant genera in the bacterial community of the four samples (Figure 6(c)). The results provide a deeper understanding of how the bacterial community changes over time. The relative abundance of *Propionivibrio* was significantly higher than that of the CON sample (Figure 6(c)). In the Aw7 sample, the abundance of *Pseudomonas, Tolumonas*, and *Aeromonas* increased compared to the other samples. In contrast, the abundances of *Longilinea*, *Bellitina*, *Dechloromonas*, *Clostridium*, *Spirochaeta*, *Syntrophus*, *Desulfobulbus*, and *Smithella* were lower compared to the other samples. Changes in these microbial abundances may be an important factor in the degradation of NH_3_-N.

## 4. Discussion

In this study, we screened NH_3_-N degrading bacteria from the river sediment of black-odor water. From these, we selected the GW1 strain, which had the highest degradation rate, for subsequent experiments. Morphological, DNA sequencing and phylogenetic tree analysis confirmed that the strain was *P. kudriavzevii. P. kudriavzevii* is commonly used in food processing [21]. To the best of our knowledge, the NH_3_-N degradation ability of *P. kudriavzevii* has not been previously reported. In this study, we identified a strain of *P. kudriavzevii* with the ability to degrade NH_3_-N. Single-factor and orthogonal experiments showed that the optimal conditions to improve the black-odor water were a *P. kudriavzevii* strain concentration of 0.3%, gas volume of 1.0 L/min, and a pH of 7.5. The optimal degradation conditions were then tested in a simulated environment. Although there will be some discrepancies with the natural environment, this study provides new insights into the application of this compound microbial agent for the bioremediation of black-odor water. Specifically, the isolated bacteria have the ability to degrade NH_3_-N from the sediment of black and odorous sewage river water, which lays a foundation for bioremediation in these areas.

Microorganisms can affect colonies in the environment by secreting some extracellular and intracellular enzymes [15]. In addition, the cell walls of some yeasts have a certain absorption ability, which can absorb microorganisms and toxins in the environment to affect the colony [22, 23]. In this experiment, we did not study the degradation mechanism of *Pichia kudriavzevii* GW1, which will be included in our subsequent experiments. In situ bioremediation with indigenous microorganisms is an effective method to eliminate pollutants [24]. Because strain GW1 was isolated from the sediment of a black and odorous sewage river, it may be more suitable for local bioremediation. 16S rRNA sequencing showed that Proteobacteria, Bacteroidetes, Chloroflexi, and Firmicutes were the main phyla in the samples. Proteobacteria and Bacteroidetes are ubiquitous in soils, wastewater, and sediments [25–28]. These results are similar to those previously obtained for the composition of bacteria in bioelectrochemically assisted constructed wetlands for treating synthetic wastewater [29, 30]. The phylum Proteobacteria contains a large number of functional aerobic bacteria that can remove organic matter and nitrogen [31]. The sclerenchyma content in the samples treated with compound microbial agents for 7, 14, and 21 days was higher than that in the control group. Previous studies have shown that Proteobacteria can reduce nitrate and perform denitrification under hypoxic conditions [32]. The increase in the amount of Proteobacteria may be a major factor in the degradation of NH_3_-N in black-odor water. At the genera level, Betaproteobacteria and Deltaproteobacteria were less abundant in the control compared to the other samples. Previous studies have reported that Betaproteobacteria and Deltaproteobacteria play a crucial role in removing nitrate and nitrite [33–35], which indicates that the relative abundance of these bacteria is closely related to the degradation of NH_3_-N in the black-odor water. The heatmap also showed that after the compound microbial agent was added, the bacterial diversity increased with time, thereby suggesting that the compound microbial agent may affect the diversity of the bacteria. Compared with the control group, the richness of *Propionivibrio, Geobacter, Pseudaeromonas, Aeromonas*, and *Tolumonas* increased significantly after 7, 14, and 21 days. A recent study showed that, at low temperatures, *Propionivibrio* was a key member of the bacterial community that simultaneously removed nitrogen and phosphorus [36]. Therefore, we also boldly predict that *Propionivibrio* plays an important role in the degradation of black-odor water; however, this hypothesis needs further verification.

## 5. Conclusion

In summary, a strain with the ability to degrade NH_3_-N has been successfully isolated from river sediments of black-odor water. This strain was identified as *Pichia kudriavzevii* GW1 based on phylogenetic analysis and phenotypic characteristics. This is the first report of a *Pichia* strain possessing the ability to degrade NH_3_-N. The optimal conditions under which strain GW1 improves black-odor water were determined. This work may be used to help develop a cost-effective and environmentally friendly method for bioremediation of black-odor water.

## Figures and Tables

**Table 1:** Growth of 12 strains in three media.

		W1	W2	W3	W4	W5	GW1	GW2	GW3	GW4	GW5	GW6	GW7
Beef extract peptone agar medium	1 d	−	−	−	−	−	−	−	−	−	−	−	−
	2 d	++	+	+	−	−	−	−	−	−	−	−	−
	3 d	+++	++	++	+	−	−	−	−	−	−	−	−

Martin's agar medium	1 d	−	−	−	−	−	−	−	−	−	−	−	−
	2 d	−	−	−	−	−	++	++	−	+	+	−	−
	3 d	−	−	−	−	−	++++	+++	+	+	+	+	+

Gao's No.1 agar medium	1 d	−	−	−	−	−	−	−	−	−	−	−	−
	2 d	−	−	−	−	−	−	−	−	−	−	−	−
	3 d	−	−	−	−	−	−	−	−	−	−	−	−

Note: “+” stands for the colony number; “−” stands for the aseptic colony.

**Table 2:** Factor and level table.

Level	*A*	*B*	*C*	*D*
	Concentration (%)	Aeration (L/min)	pH	Temperature (°C)
1	0.1	0.5	6.5	30
2	0.2	1.0	7.0	35
3	0.3	1.5	7.5	40

**Table 3:** Orthogonal design for NH_3_-N removal rate and COD removal rate.

No.	*A*	*B*	*C*	*D*	NH_3_-N removal rate (%)	COD removal rate (%)
	Concentration	Aeration	pH	Temperature		
1	1	1	1	1	77	(78)
2	1	2	2	2	82	(84)
3	1	3	3	3	81	(82)
4	2	1	2	3	80	(84)
5	2	2	3	1	84	(84)
6	2	3	1	2	90	(91)
7	3	1	3	2	93	(86)
8	3	2	1	3	84	(77)
9	3	3	2	1	94	(88)
*K*1	80 (81.3)	83.3 (82.7)	83.7 (82)	85 (83.3)	—	—
*K*2	84.7 (86.3)	83.3 (81.7)	85.3 (85.3)	88.3 (87.7)	—	—
*K*3	90.3 (83.7)	88.3 (87)	86 (84)	83 (81)	—	—
*R*	10.3 (5)	5 (5.3)	2.3 (3.3)	5.3 (6.7)	—	—

## Data Availability

The data used to support the findings of this study are available from the corresponding author upon request.
